# The Potential of Use Basil and Rosemary Essential Oils as Effective Antibacterial Agents

**DOI:** 10.3390/molecules18089334

**Published:** 2013-08-05

**Authors:** Monika Sienkiewicz, Monika Łysakowska, Marta Pastuszka, Wojciech Bienias, Edward Kowalczyk

**Affiliations:** 1 Medical and Sanitary Microbiology Department, Medical University of Lodz, pl. Hallera 1, Lodz 90-647, Poland; E-Mail: monika.lysakowska@umed.lodz.pl; 2 Department of Dermatology, Pediatric Dermatology and Oncology, Medical University of Lodz, Kniaziewicza 1/5, Lodz 91-347, Poland; E-Mail: dermatologia@umed.lodz.pl (M.P. & W.B.); 3 Pharmacology and Toxicology Department, Medical University of Lodz, Pl. Hallera 1, Lodz 90-647, Poland; E-Mail: edward.kowalczyk@umed.lodz.pl

**Keywords:** basil oil, ESBL-positive strains, *Escherichia coli*, minimal inhibitory concentration, rosemary oil

## Abstract

The considerable therapeutical problems of persistent infections caused by multidrug-resistant bacterial strains constitute a continuing need to find effective antimicrobial agents. The aim of this study was to demonstrate the activities of basil (*Ocimum basilicum* L.) and rosemary (*Rosmarinus officinalis* L.) essential oils against multidrug- resistant clinical strains of *Escherichia coli*. A detailed analysis was performed of the resistance of the drug to the strains and their sensitivity to the tested oils. The antibacterial activity of the oils was tested against standard strain *Escherichia coli* ATCC 25922 as well as 60 other clinical strains of *Escherichia coli*. The clinical strains were obtained from patients with infections of the respiratory tract, abdominal cavity, urinary tract, skin and from hospital equipment. The inhibition of microbial growth by both essential oils, presented as MIC values, were determined by agar dilution. Susceptibility testing to antibiotics was carried out using disc diffusion. The results showed that both tested essential oils are active against all of the clinical strains from *Escherichia coli* including extended-spectrum β-lactamase positive bacteria, but basil oil possesses a higher ability to inhibit growth. These studies may hasten the application of essential oils in the treatment and prevention of emergent resistant strains in nosocomial infections.

## 1. Introduction

The multidrug-resistant pathogenic strains of *Escherichia coli* are responsible for opportunistic infections, including nosocomial ones, which are difficult to treat, especially in immunocompromised patients. *E. coli* is responsible for severe cases of urinary tract infection, meningitis in newborns, digestive system illnesses, and even pneumonia. In recent years, strains of *Enterobacteriaceae* producing an extended spectrum β-lactamase have become a concern in the antimicrobial treatment of persistent infections and control of infection in hospitals [1,2,3,4,5]. The most severe clinical cases are isolated resistant strains of *Escherichia coli*, *Klebsiella pneumonia*, *Pseudomonas aeruginosa* and *Acinetobacter baumanii*. Extended-spectrum β-lactamases are enzymes produced by Gram-negative bacilli that mediate resistance to penicillin, cephalosporins, and monobactams [6,7,8,9]. The widespread use of antimicrobial drugs, primarily antibiotics, and the transmissibility of resistance determinants mediated by plasmids, transposons, and gene cassettes in integrons contribute to the spread of resistance.

A worrying development is the fast spread of resistant clones of these bacteria on a global scale [10,11,12]. Thanks to growing resistance to drugs commonly used in clinical practice, effective treatment availability is greatly reduced. This problem of increasing resistance has necessitated the search for safe and effective factors that may be used to treat persistent bacterial infections. Experimental research confirms the varied pharmaceutical activities of not only chemical compounds, but also many plant metabolites such as polysaccharides, flavonoids, coumarins, glycosides, phenolic acids, saponins and also essential oils. Plant metabolites are a very interesting alternative for synthetic preparations: many of them have strong antimicrobial activity [13,14,15,16,17]. Their synergy of action with each other and in combination with antibiotic and chemotherapeutic therapy make them a valued complement to anti-infective therapy [18,19,20,21,22,23].

The *Ocimum* L. (basil) and *Rosmarinus* L. (rosemary) genera belong to the family *Lamiaceae*. Among the plants known for medicinal value, basil and rosemary plants are highly regarded for their therapeutic potentials. *Ocimum basilicum* L. and *Rosmarinus officinalis* L. essential oils offer promise as biologically active constituents, in that they confer antibacterial, antifungal and antioxidant properties. Basil and rosemary oils have long been used for treating, among other things, colds, fever, cough, asthma, sinusitis and rheumatism, as well as accelerating the process of wound healing [24,25,26,27,28]. The aim of this work was to determine the antibacterial activity of basil oil from *Ocimum basilicum* L. and rosemary oil from *Rosmarinus officinalis* L. against standard and clinical strains of *Escherichia coli* isolated from patients and from hospital equipment.

## 2. Results

### 2.1. Chemical Composition of the Basil and Rosemary Essential Oils

Chemical analysis showed the presence of forty-eight constituents within basil oil from *Ocimum basilicum* L., the main ones being estragole (86.4%), 1,8-cineole (4.9%) and *trans*-α-bergamotene (3.0%). The chemical composition of the basil oil is presented in Table 1. The essential oil from *Rosmarinus officinalis* L. contains thirty-seven components: the main ones being 1,8 cineole (46.4%), camphor (11.4%), α-pinene (11.0%), β-pinene (9.2%) and camphene (5.2%). The chemical composition of the rosemary oil is presented in Table 2.

**Table 1:** Constituents of basil oil.

Number of compounds	Compound	%		RI	
1	α-Pinene	0.4		929	
2	Camphene	0.1		942	
3	Sabinene	0.2		965	
4	β-Pinene	0.6		969	
5	2,3-Dehydro-1.8-cineole	Tr		979	
6	Myrcene	0.2		983	
7	*p*-Cymene	0.1		1013	
**8**	**1,8-Cineole**	**4.9**		**1020**	
9	Limonene	0.4		1021	
10	(E)-β-Ocimene	0.6		1038	
11	trans-Linalool oxide (f)	Tr		1058	
12	Fenchone	0.2		1067	
13	cis-Linalool oxide (f)	Tr		1073	
14	Linalool	1.2		1085	
15	endo-Fenchol	0.2		1098	
16	Camphor	0.7		1119	
17	Menthone	0.1		1134	
18	Isomenthone	Tr		1143	
19	Borneol	0.2		1149	
20	Menthol	0.3		1159	
21	Terpinen-4-ol	0.1		1163	
**22**	**Estragole**	**86.4**		**1188**	
23	Fenchyl acetate	0.3		1209	
24	Bornyl acetate	0.3		1269	
25	2-Hydroxycineol acetate	Tr		1321	
26	Eugenol methyl ether	0.5		1373	
27	β-Bourbonene	Tr		1385	
28	β-Elemene	0.3		1389	
29	cis-α-Bergamotene	Tr		1412	
30	β-Caryophyllene	0.1		1419	
**31**	**trans-α-Bergamotene**	**3.0**		**1435**	
32	β-Sesquifenchene	0.2	1437		
33	(Z)-β-Farnesene	tr	1447		
34	α-Humulene	tr	1452		
35	Cadina-1(6),4-diene	tr	1459		
36	trans-β-Bergamotene	0.2	1479		
37	α-Bulnesene	0.1	1499		
38	γ-Cdinene	0.5	1506		
39	Calamenene	tr	1510		
40	β-Sesquiphellandrene	0.1	1514		
41	*p*-Methoxycinnamaldehyde	0.5	1525		
42	*p*-Methoxycinnamyl alcohol	0.4	1532		
43	Spathulenol	0.1	1565		
44	Caryophyllene oxide	0.1	1571		
45	Humulene epoxide II	0.1	1595		
46	1- *epi*-Cubenol	0.1	1603		
47	T-Cadinol	0.7	1627		
48	α-Cadinol	tr	1639		

RI-Retence Index; tr < 0.05%.

**Table 2:** Constituents of rosemary oil.

Number of compounds	Compound	%	RI
1	Tricyclene	0.2	919
2	α-Thujene	0.1	923
**3**	**α-Pinene**	**11.0**	**932**
**4**	**Camphene**	**5.2**	**944**
5	Sabinene	0.1	966
**6**	**β-Pinene**	**9.2**	**971**
**7**	**Myrcene**	**1.2**	**983**
8	α-Phellandrene	0.2	997
9	Car-3-ene	0.1	1005
10	α-Terpinolene	0.1	1010
11	*p*-Cymene	1.3	1017
**12**	**1,8-Cineole**	**46.4**	**1027**
**13**	**Limonene**	**1.0**	**1027**
14	γ-Terpinene	1.0	1050
15	*trans*-Sabinene	tr	1054
16	Terpinolene	0.2	1079
17	Linalool	0.5	1087
18	α-Campholenol	tr	1096
19	*endo*-Fenchol	tr	1102
**20**	**Camphor**	**11.4**	**1124**
**21**	**Borneol**	**3.1**	**1152**
22	Terpinen-4-ol	0.4	1163
**23**	**α-Terpineol**	**1.8**	**1175**
**24**	**Bornyl acetate**	**1.0**	**1269**
25	α-Cubebene	tr	1349
26	α-Ylangene	tr	1372
27	α-Copaene	0.1	1377
28	Longifolene	0.1	1407
**29**	**β-Caryophyllene**	**3.5**	**1421**
30	α-Humulene	0.4	1452
31	γ-Muurolene	0.1	1471
32	α-Selinene	tr	1492
33	α-Muurolene	tr	1494
34	γ-Cadinene	tr	1506
35	*trans*-Calamenene	tr	1511
36	δ-Cadinene	0.1	1514
37	β-Caryophyllene oxide	0.1	1571

RI-Retention Index; tr < 0.05%.

### 2.2. Susceptibility Testing of Clinical *Escherichia coli* Strains

#### 2.2.1. Susceptibility Testing of Clinical *Escherichia coli* (ESBL+) Strains

**Figure 1:**
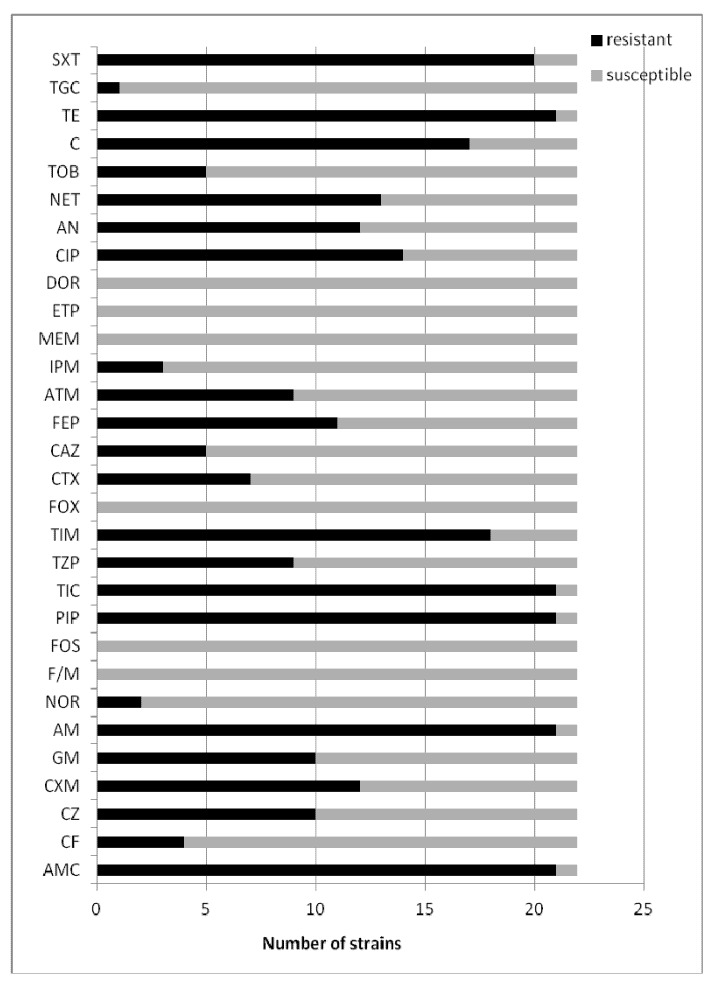
The susceptibility testing of clinical *Escherichia coli* (ESBL+) strains.

Extended spectrum β-lactamase production for the tested *Escherichia coli* clinical strains was detected for strains from the abdominal cavity (n = 4), bronchia (n = 4), wounds (n = 4), urine (n = 4) and for strains isolated from blood (n = 3) and catheters (n = 3). The results are shown in Figure 1. The tested strains of *Escherichia coli* (ESBL+) were generally resistant to β-lactams, aminoglycosides and quinolones recommended for susceptibility testing. Most of them were resistant to cephalosporins and β-lactam antibiotics with such inhibitors as clavulanic acid, sulbactam and tazobactam.

#### 2.2.2. Susceptibility Testing of Clinical *Escherichia coli* (ESBL−) Strains

**Figure 2:**
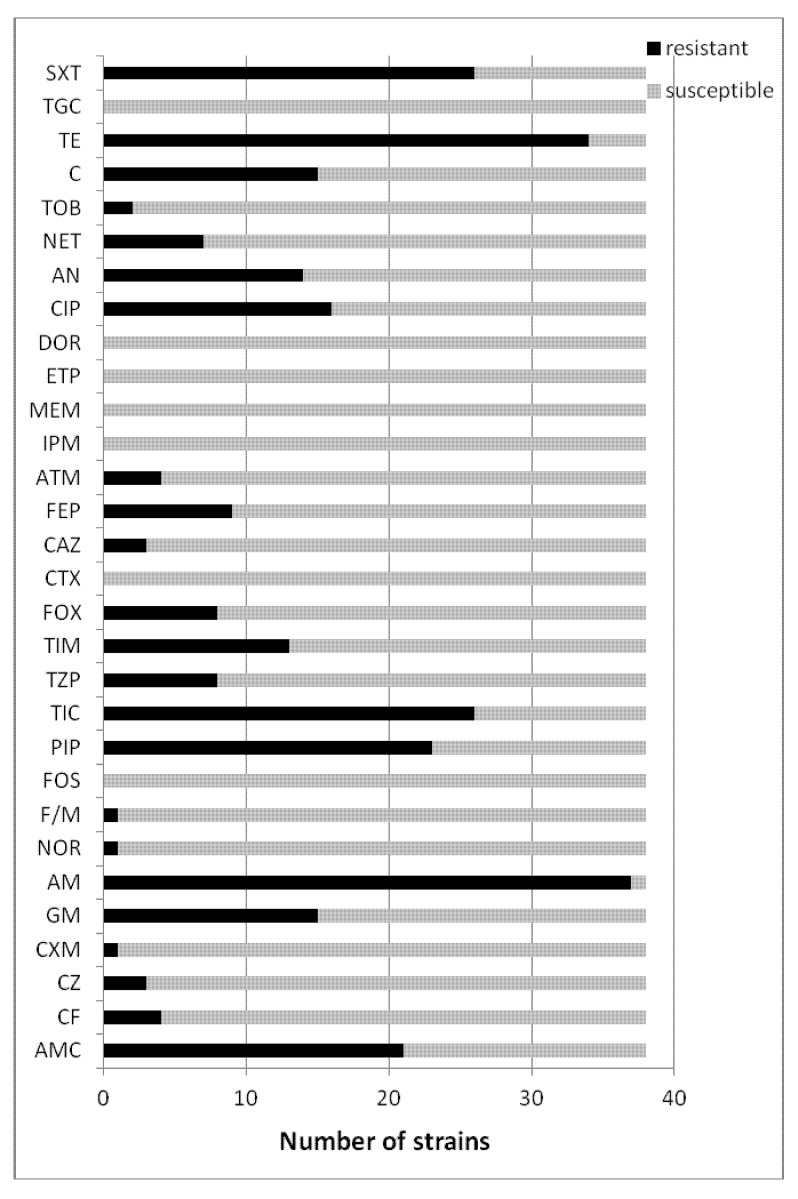
The susceptibility testing of clinical *Escherichia coli* (ESBL−) strains.

*Escherichia coli* ESBL negative strains, characterized by a much lower resistance to β-lactam antibiotics, were resistant mainly to ampicillin, piperacillin, tikarcillin and also to ticarcillin/clavulanic acid. Most of them were resistant to aminoglycosides (gentamicin, amikacin) and quinolones (ciprofloksacin) and tetracycline. The results are shown in Figure 2.

The (ESBL+) and (ESBL−) *Escherichia coli* were found to demonstrate significant resistance to the reference antibiotics in the susceptibility tests. In our tests, all twenty-two of the tested clinical isolates of ESBL positive *E. coli* were resistant to AMC (n = 21, 95%), CZ (n = 10, 45%), CXM (n = 12, 54%), GM (n = 10, 45%), AM (n = 21, 95%), PIP (n = 21, 95%), TIC (n = 21, 95%), TIM (n = 18, 81%), FEP (n = 11, 50%), CIP (n = 14, 64%), AN (n = 12, 54%), NET (n = 13, 59%), C (n = 17, 77%), TE (n = 21, 95%) and SXT (n = 20, 91%). The thirty-eight tested ESBL negative *E. coli* strains were generally resistant to AMC (n = 21, 55%), GM (n = 15, 39%), PIP (n = 23, 60%), TIC (n = 26, 68%), TIM (n = 13, 34%), CIP (n = 16, 42%), AN (n = 14, 37%), C (n = 15, 39%), TE (n = 34, 89%) and SXT (n = 26, 68%).

### 2.3. The Susceptibility *Escherichia coli* Bacterial Strains to Basil Oil

The MIC values for sixty tested *E. coli* strains were between 8.0 µL/mL to 11.5 µL/mL. The basil oil showed inhibitory activity against *E. coli* ATCC 25922 standard strain at 8.0 µL/mL.

#### 2.3.1. The Susceptibility *Escherichia coli* (ESBL+) Strains to Basil Oil

**Figure 3:**
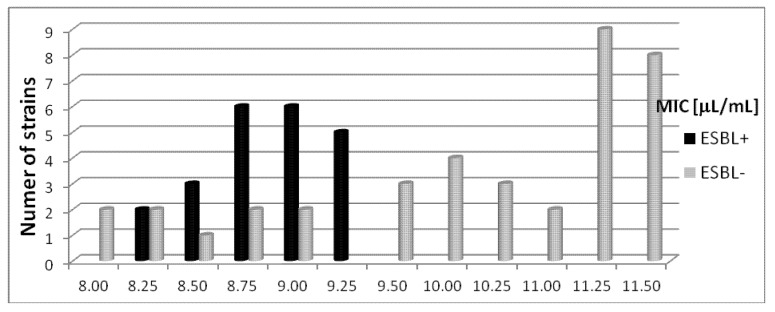
The susceptibility of *Escherichia coli* (ESBL+) and (ESBL−) strains to basil oil.

Most *E. coli* ESBL+ strains isolated from the abdominal cavity (n = 4) and from the bronchia (n = 4) were sensitive to basil oil at a concentration range from 8.25 µL/mL to 9.0 µL/mL. For (ESBL+) clinical strains from wounds (n = 4), MIC values were between 8.5 µL/mL to 9.25 µL/mL. The growth inhibition concentrations for bacteria isolated from blood were 8.75 µL/mL (n = 1) and 9.25 µL/mL (n = 2). Basil oil at a concentration of 8.75 µL/mL inhibited the growth of one of the tested clinical strains isolated from urine, while 9.0 µL/mL inhibited the growth of three. The MIC values for strains from catheters were 8.75 µL/mL (n = 2) and 9.25 µL/mL (n = 1). The susceptibility of *Escherichia coli* (ESBL+) strains to basil oil is shown in Figure 3.

#### 2.3.2. The Susceptibility *Escherichia coli* (ESBL−) Strains to Basil Oil

The basil oil was active against *Escherichia coli* (ESBL−) strains at a concentration range from 8.25 µL/mL to 11.5 µL/mL. The most tested strains isolated from the abdominal cavity (n = 4) were inhibited by concentrations of 11.0–11.5 µL/mL. Clinical strains from the bronchia (n = 5) were sensitive to basil oil at concentrations of 10.25–11.5 µL/mL. The MIC values for most strains from wounds (n = 4) were from 10.0 µL/mL to 11.5 µL/mL. Most *Escherichia coli* (ESBL−) strains isolated from blood and urine were inhibited by basil oil at concentrations ranging from 9.5 µL/mL to 11.5 µL/mL. The MIC values for bacteria from the catheters (n = 6) were 10.0–11.5 µL/mL. The susceptibility of *Escherichia coli* (ESBL−) strains to basil oil is demonstrated in Figure 3.

### 2.4. The Susceptibility *Escherichia coli* Bacterial Strains to Rosemary Oil

The rosemary oil was less active against the sixty tested *Escherichia coli* clinical strains obtained from the diverse clinical materials and the hospital equipment. The MIC values were between 18.0 and 20.0 µL/mL. The standard strain *E. coli* ATCC 25922 was sensitive to rosemary oil at a concentration of 18.5 µL/mL.

#### 2.4.1. The Susceptibility of *Escherichia coli* (ESBL+) Strains to Rosemary Oil

**Figure 4:**
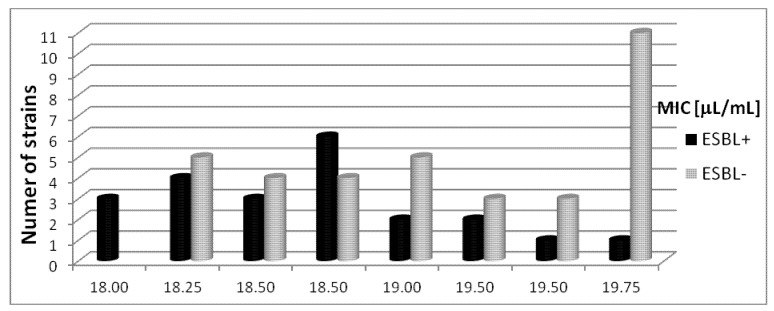
The susceptibility of *Escherichia coli* (ESBL+) and (ESBL−**)** strains to rosemary oil.

Most *Escherichia coli* (ESBL+) strains isolated from the abdominal cavity (n = 3) were sensitive to rosemary oil at concentrations from 18.0 µL/mL to 18.5 µL/mL, and (n = 1) at 19.25 µL/mL concentration. The MIC values for bacterial strains isolated from the bronchia (n = 4) and wounds (n = 4) were 18.25–19.0 µL/mL and 18.5–19.25 µL/mL, respectively. *E. coli* (ESBL+) strains isolated from blood were sensitive to rosemary oil at concentrations between 18.75 and 19.75 µL/mL. All strains from urine (n = 4) were inhibited in the concentration ranges 18.0 µL/mL to 18.75 µL/mL, while those from catheters (n = 3) were inhibited from 18.25 µL/mL to 18.75 µL/mL. The results of the tests detailing the susceptibility of *Escherichia coli* (ESBL+) strains to rosemary oil are presented in Figure 4.

#### 2.4.2. The Susceptibility of *Escherichia coli* (ESBL−) Strains to Rosemary Oil

Rosemary oil was found to have MIC values from 18.5 µL/mL to 19.75 µL/mL for isolates from the abdominal cavity (n = 6). Growth inhibition was found to occur at concentrations of 19.0–20.0 µL/mL for clinical bacterial strains obtained from the bronchia (n = 6). Rosemary oil at concentrations ranging from 18.25 µL/mL to 20.0 µL/mL inhibited the growth of all tested *Escherichia coli* (ESBL−) strains isolated from wounds. For the blood (n = 7) and urine (n = 6) isolates, the MIC values were in the range 18.25-19.75 µL/mL. Finally, similar MIC values were found (18.25–19.75 µL/mL) against *E. coli* (ESBL−) strains isolated from catheters (n = 7). The susceptibility of the *Escherichia coli* (ESBL−) strains to rosemary oil is shown in Figure 4.

## 3. Discussion

In our investigation, all clinical strains of *Escherichia coli* were found to be sensitive to basil (*Ocimum basilicum* L.) and rosemary (*Rosmarinus officinalis* L.) essential oils, irrespective of the clinical conditions they were obtained under or the pattern of antibiotic resistance they demonstrated, but basil oil was more active against the tested bacteria. Out of the sixty clinical strains of *E. coli*, twenty-two strains were extended-spectrum beta-lactamase positive (ESBL+). Concentrations of basil oil ranging from 8.25 µL/mL to 9.25 µL/mL were seen to inhibit the growth of eighteen *Escherichia coli* (ESBL+) strains. For the extended-spectrum beta-lactamase negative strains, seventeen out of the thirty-eight were sensitive to basil oil at concentrations between 11.25–11.50 µL/mL. The MIC values for the other twenty-one *Escherichia coli* (ESBL−) strains ranged from 8.0 µL/mL to 11.0 µL/mL.

The rosemary oil demonstrated significantly lower activity. No apparent differences in activity of the essential oil were found against extended-spectrum β-lactamase-positive and negative strains. Out of the twenty-two clinical strains of *E. coli* (ESBL+), rosemary oil concentrations ranging from 18.0 µL/mL to 19.0 µL/mL were effective for eighteen of them. Eleven of the *Escherichia coli* (ESBL−) strains out of the thirty-eight obtained under various conditions were inhibited by 19.75 µL/mL of rosemary oil. MIC values ranging from 18.25 µL/mL to 19.5 µL/mL for comparable numbers of strains was obtained.

The results of our tests clearly demonstrate that basil and rosemary essential oils can be widely used to eliminate clinical strains of *Escherichia coli* found in different clinical conditions. It is also significant that extended-spectrum β-lactamase (ESBL)− producing clinical strains of *E. coli* are sensitive to these oils. Studies Orhan at al [29], confirm that, essential oils from *Foeniculum* sp., *Mentha* sp., *Ocimum* sp., *Origanum *sp. and *Satureja* sp. (*Lamiaceae* family) possess strong antibacterial activity against extended-spectrum beta-lactamase (ESBL) positive strains of *Klebsiella pneumoniae* isolated from food. The obtained MIC values ranged from 32 to 64 μg/ml for a number of strains resistant to trimetoprime-sulfametoxazole, sulbactam-ampicilin, clavulonate-amoxicilin, ceftriaxon, cefepime, imipenem, ceftazidime, tobramicine, gentamisine, ofloxacin, and ciprofloxacin. According our investigations, the *Escherichia coli* (ESBL+) responsible for human infectious diseases were significantly more resistant to the basil and rosemary essential oils.

The antimicrobial properties of essential oils are strictly connected with their chemical composition. The usefulness of essential oils as effective antimicrobial agents can be evaluated only by analysing their individual components. Therefore, a thorough GC-FID-MS analysis of the tested basil and rosemary essential oils was conducted. The composition of the essential oil obtained from *Ocimum basilicum* L., is as given in the ISO-11043 standard. The composition of the tested basil oil corresponded to required standards, according to which the content of estragole must be higher than 75.0%. The composition of the essential oil derived from *Rosmarinus officinalis* L. was found to meet the requirements of the European Pharmacopoeia 6 [30] and of the Polish Pharmacopoeia VIII [31] for the nine main components. The content of β-pinene amounted to 9.2% (required 4.0%–9.0%) and limonene to 1.0% (required 1.5%–4.0%). Verbenone was not found among the components of the tested rosemary oil, although EP 6 and the Polish Pharmacopoeia VIII specify its content to be a maximum of 0.4%. For the tested rosemary essential oil, nine of the thirteen main constituents of the oil met the requirements given in the ISO-1342 standard: α-pinene, camphene, myrcene, 1,8-cineole, *p*-cymene, camphor, bornyl acetate, α-terpineol and borneol.

The highest antibacterial activity is demonstrated by phenolic compounds such as carvacrol, thymol and eugenol. Another effective group of active compounds are alcohols: terpinen-4-ol, γ-terpineol, geraniol, cytronellol, menthol and linalol. Many of them are synthesized by plants from the *Lamiaceae* family [32]. For instance, the essential oil of *Satureja hortensis* L. demonstrates high levels of activity. Mihajilov-Kristev *et al*. [33], showed that essential oil containing mainly carvacrol (67.0%) and γ-terpinene (15.3%) is effective against Gram-negative strains, including *Escherichia coli*, with MIC values from 0.025 µL/mL to 0.78 µL/mL according to the broth microdilution method. In our study for basil and rosemary essential oils, we obtained significantly higher MIC values. This high activity demonstrated by *Satureja hortensis* L. essential oil is certainly related to the high content of carvacrol, which is one of the most potent antimicrobial compounds.

The literature reports that basil oil, which contains mainly estragole and linalool, also possesses antibacterial agents which are effective against a variety of Gram-positive and Gram-negative bacteria [34]. According to Saković *et al*. [35], *Ocimum basilicum* essential oil possesses antibacterial activity against the human pathogenic bacteria *Bacillus subtilis*, *Enterobacter cloacae*, *Escherichia coli* O157:H7, *Micrococcus flavus*, *Proteus mirabilis*, *Pseudomonas aeruginosa*, *Salmonella enteritidis*, S. *typhimurium*, *Staphylococcus epidermidis* and *S. aureus*. According to the authors, basil essential oil containing 69.3% linalool as a main component possesses antibacterial properties against *Escherichia coli* O157:H7 with MIC and MBC values of 6.0 µg/mL when assessed by microdilution. The multidrug clinical strains of *E. coli* tested in this study were more resistant to basil essential oil containing mainly estragole (86.4%).

Results obtained by Sartoratto *et al*. [36], show that basil oil from *Ocimum basilicum* containing mainly linallol 32.6% and eugenol 28.1%, and oil from *Ocimum gratissimum* containing 93.9% eugenol, have a broad spectrum of antibacterial activity against reference strains of Gram-positive, Gram-negative bacteria and *Candida albicans*. According to the authors, essential oils obtained from these two genera of *Ocimum* were active against *Escherichia coli* CCT0547 standard strain with a MIC value of >2 mg/mL according to the microplate method. These results also confirm that oils with active constituents such as eugenol tend to have high antibacterial properties. Our results are slightly higher than those obtained by Sartoratto *et al*. The MIC values were in the range from 7.92 mg/mL to 11.04 mg/mL for clinical strains of *Escherichia coli* which were both positive and negative for extended-spectrum beta-lactamase activity.

Nakamura *et al*. [37], demonstrated that the essential oil from *Ocimum gratissimum,* with eugenol as its main constituent, possesses antibacterial activity against clinical strains of *Escherichia coli* ATCC 25922 with MIC—6 mg/mL. The MICs of this essential oil against a group of other Gram-negative bacteria comprising *Shigella flexneri*, *Salmonella enteritidis*, *Klebsiella* sp. and *Proteus mirabilis* were found to be from 0.3 to 12 mg/mL. The MIC values given in our study were slightly higher than those given by Nakamura *et al*. Pereira *et al*. [10], in their investigations, showed that oil from *Ocimum gratissimum* possesses antibacterial activity against clinical *Escherichia coli* strains isolated from urinary tract infections. The essential oil was found to be active against about 70% of tested *E. coli* clinical isolates. The results of the present study demonstrate that a chemotype containing mainly 1,8-cineole, eugenol, methyleugenol, thymol, *p*-cimene, *cis*-ocimene and *cis*-caryophyllene has lower activity.

In our tests, basil oil obtained from *Ocimum basilicum,* containing mainly estragole (86.4%), inhibited the growth all strains isolated from various clinical materials. Among them were bacteria isolated from urine, which were also extended-spectrum beta-lactamase positive. Our studies confirm that antibacterial activity is possessed by not only basil oil chemotypes with linalool or eugenol as their main components, but also that of *Ocimum basilicum,* containing mainly estragole. Our research showed that basil essential oil was significantly more effective against all clinical isolates than rosemary essential oil. Similar results were obtained by Hammer *et al* [38], who studied the antimicrobial activity of basil and rosemary essential oils against *Acinetobacter baumanii*, *Aeromonas veronii* biogroup *sobria*, *Candida albicans*, *Enterococcus faecalis*, *Escherichia coli*, *Klebsiella pneumoniae*, *Pseudomonas aeruginosa*, *Salmonella enterica* subsp. *enterica* serotype *typhimurium*, *Serratia marcescens* and *Staphylococcus aureus*, using an agar dilution method. The authors confirmed that basil (*Ocimum basilicum*) oil is more active against *Escherichia coli* than rosemary (*Rosmarinus officinalis*). The MIC values were 0.5 and 1.0% (*v*/*v*), respectively.

According to Lopez *et al*. [39], the oils from *Ocimum basilicum* and *Rosmarinus officinalis* have an antibacterial potential against the Gram-positive bacteria *Staphylococcus aureus*, *Enterococcus faecalis* and *Listeria monocytogenes* and against Gram-negative bacteria *Escherichia coli*, *Yersinia enterocolitica*, *Salmonella choleraesuis* and *Pseudomonas aeruginosa* as foodborne bacterial strains. The authors present a detailed analysis of the tested oils and their ability to inhibit the growth of bacteria. Their basil and rosemary essential oils were of a similar composition to the essential oils in our investigations. The main component of the basil essential oil was estragole—82% ± 1.2%, while the rosemary essential oil contains 1.8-cineole—48% ± 9.1%, camphor—17% ± 4.0, β-pinene—4.8% ± 0.9% and β-caryophyllene—6.8% ± 3.7%. The authors confirm that the basil oil is more effective at inhibiting the growth of *Escherichia coli* strains.

Probuseenivasan *et al* [27], confirmed that rosemary essential oil strongly inhibits *Escherichia coli* ATCC 25922. Although the basil oil was also seen to demonstrate low activity against the tested bacteria, no data was given about the constituents of the essential oils. The minimal inhibitory concentration for rosemary oil against *E. coli* was >6.4 mg/mL. The MIC values obtained by the present study were higher and ranged from 16.02 mg/mL to 17.35 mg/mL against *E. coli* clinical strains. Fabio *et al* [40], report that rosemary oil has an antibacterial effect on a number of microorganisms responsible for respiratory infections, isolated from clinical specimens, among which were antibiotic-sensitive and antibiotic-resistant strains such as *Streptococcus pyogenes*, *S. agalactiae*, *S. pneumoniae* and *Klebsiella pneumoniae*, *Staphylococcus aureus* and *Stenotrophomonas maltophilia*. In our tests, rosemary oil was also found to demonstrate antibacterial activity against *Escherichia coli* strains with different patterns of resistance, including extended-spectrum β-lactamase positive strains isolated from various clinical materials. The rosemary essential oil used in the present study obtained from *Rosmarinus officinalis* contains mainly 1,8-cineole (46.4%), camphor (11.4%) and α-pinene (11.0%). The composition of the rosemary essential oil used by Jiang *et al.* [41], was similar to that used by us: mainly 1,8-cineole (26.54%) and α-pinene (20.14%). The authors show that the tested oil possesses antibacterial activity against Gram-positive bacteria (*Staphylococcus epidermidis*, *Staphylococcus aureus* and *Bacillus subtilis*), Gram-negative bacteria (*Proteus vulgaris*, *Pseudomonas aeruginosa* and *Escherichia coli*) and fungi (*Candida albicans* and *Aspergillus niger*). Bendeddouche *et al* [42], showed that of essential oil from *Rosmarinus tournefortii* De Noé growing wild in the occidental region of Algeria possesses antimicrobial activity also against Gram-negative (*Escherichia coli* and *Pseudomonas aeruginosa*) and Gram-positive (*Staphylococcus aureus*) pathogenic bacteria. The main constituents of the tested essential oil were camphor (37.6%), 1,8-cineole (10.0%), *p*-cymene-7-ol (7.8%) and borneol (5.4%).

A number of studies show that essential oils and their constituents possess useful properties concerning human health. Many of them may be applied in anticancer therapy, cardiovascular and nervous system disorders to reduce the level of cholesterol, to regulate the glucose level or to stimulate hormone production [43]. They also might have great value in preventing and treating infectious diseases. Essential oils not only have bactericidal activity but also can inhibit multidrug bacterial strain formation. Their multiple antibacterial, antifungal, antiviral and also anti-inflammatory and antioxidant effects, have made them valuable agents in human treatment and for the prevention of pathological changes. In addition, essential oils have a number of beneficial properties as natural preservatives in cosmetics, toiletries, drugs and food products [44,45,46]. Considering the huge increase in the number of multidrug resistant bacterial strains in health care facilities, essential oils may prove to be effective natural antimicrobial agents.

## 4. Experimental

### 4.1. Bacterial Strains

The standard strain, *E. coli* ATCC 25922, was obtained from the collection of the Medical and Sanitary Microbiology Department, Medical University of Lodz. The clinical strains of *Escherichia coli* were collected in 2011 and 2012 from a range of clinical materials recovered from patients and from the hospital equipment in various wards from one of the Medical University hospitals in Lodz: internal medicine, surgery, urology and the intensive care unit. The tested bacterial strains were isolated from the abdominal cavity (n = 10), bronchia (n = 10), wounds (n = 10), blood (n = 10), urine (n = 10) and from catheters (n = 10).

### 4.2. Bacterial Strain Identification

*E. coli* strains were cultured on Columbia Agar (bioMerieux, Craponne, France) and on Mac Conkey Agar (bioMerieux). They were identified to the species by using API 20 E tests (bioMerieux). The bacteria were incubated at 37 °C for 24 h.

### 4.3. Essential Oil Analysis

Commercial essential oils from basil—*Ocimum basilicum* L. and rosemary—*Rosmarinus officinalis* L. were purchased from the manufacturer (POLLENA-AROMA, Warsaw, Poland) and analyzed by GC-FID-MS in the Institute of General Food Chemistry, Lodz University of Technology, using a Trace GC Ultra apparatus (Thermo Fisher Scientific Inc., Waltham, MA, USA) MS DSQ II detectors and FID-MS splitter (SGE). Operating conditions: apolar capillary column Rtx-1ms (Restek Corporation, Bellefonte, PA, USA), 60 m × 0.25 mm i.d., film thickness 0.25 µm; temperature program, 50–300 °C at 4 °C/min; SSL injector temperature 280 °C; FID temperature 300 °C; split ratio 1:20; carrier gas helium at regular pressure 200 kPa.; FID temperature 260 °C; carrier gas, helium; 0.5 mL/min; split ratio 1:20. Mass spectra were acquired over the mass range 30–400 Da, ionization voltage 70 eV; ion source temperature 200 °C. The analysis of the constituents of the oils were performed two times independently.

Identification of components was based on the comparison of their MS spectra with those of the laboratory-made MS library, commercial libraries (NIST 98.1, Wiley Registry of Mass Spectral Data, 8th Ed. and MassFinder 3.1) and with literature data [47,48] along with the retention indices on the apolar column (Rtx-1, MassFinder 3.1) associated with a series of alkanes with linear interpolation (C_8_-C_26_). A quantitative analysis, expressed as percentages of each component, was carried out by peak area normalization measurements without correction factors.

### 4.4. Antibacterial Tests

The standard and clinical strains were cultivated in Columbia Agar medium and incubated at 37 °C for 48 h in aerobic conditions. The microbial suspension was standardized to a cell density of 1–2∙× 10^8^ cells/mL, equal to an optical density of 0.5 on the Mc Farland scale, by a bioMerieux densitometer. The agar dilution method was employed for the screening of antimicrobial activities of the essential oils [32,38,49,50,51]. The tested essential oils were diluted in 96% ethanol PURE (POCH, Gliwice, Poland) yielding a concentration of 97% *v*/*v* of oils. Although the tested essential oils dissolve well in ethanol, only minimum amounts were used, as it can inhibit the growth of the tested bacteria. This solution was mixed with a culture medium to obtain concentrations from 7.25 µL/mL to 11.75 µL/mL for basil oil and 17.75 µL/mL to 20.25 µL/mL for rosemary oil and poured into sterile Petri dishes. An inoculum containing 1–2 × 10^8^ cells/mL (0.1 mL) per spot was seeded upon the surface of the agar with various oil concentrations, as well as on agar with no oil added (acting as a control for strain growth). The Minimal Inhibitory Concentration, MIC, was determined after 24 h of incubation at 37 °C under aerobic conditions. The MIC was considered the lowest concentration of the sample at which no visible growth was observed. The analysis of the antibacterial activity of the oil was performed three times independently. Control media containing only alcohol at concentrations used in the dilutions of tested essential oils did not inhibit the growth of bacterial strains.

### 4.5. Susceptibility Testing

The following antibiotics (*Becton Dickinson*) were used for susceptibility testing of *Escherichia coli* strains (R—resistance; I—intermediate susceptibility; S—susceptibility): AM—ampicillin (10 µg) (R ≤ 13, 14 ≤ I ≤ 16, S ≥ 17), AMC—amoxicillin/clavulanic acid (20 µg/10 µg) (R ≤ 13, 14 ≤ I ≤ 17, S ≥ 18), CF—cefalotin (30 µg) (R ≤ 14, 15 ≤ I ≤ 17, S ≥ 18), CZ—cefazoline (30 µg) (R ≤ 14, 15 ≤ I ≤ 17, S ≥ 18), CXM—cefuroxime (30µg) (R ≤ 14, 15 ≤ I ≤ 17, S ≥ 18), GM—gentamicin (10 µg) (R ≤ 12, 13 ≤ I ≤ 14, S ≥ 15), TE—tetracycline (30 µg) (R ≤ 14, 15 ≤ I ≤ 18, S ≥ 19), NOR—norfloxacin (10 µg) (R ≤ 12, 13 ≤ I ≤ 16, S ≥ 17) (only for the isolates from urine), FTN—nitrofurantoin (300 µg) (R ≤ 14, 15 ≤ I ≤ 16, S ≥ 17) (as above), FOS—fosfomycin (200 µg) (R ≤ 12, 13 ≤ I ≤ 15, S ≥ 16) (as above), STX—trimethoprim/sulfamethoxazole (1.25 µg/23.75 µg) (R ≤ 10, 11 ≤ I ≤ 15, S ≥ 16), PIP—piperacillin (100 µg) (R ≤ 17, 18 ≤ I ≤ 20, S ≥ 21), TIC—tikarcillin (75 µg) (R ≤ 14, 15 ≤ I ≤ 19, S ≥ 20), TZP—piperacyllin/tazobaktam (100/10 µg) (R ≤ 17, 18 ≤ I ≤ 20, S ≥ 21), TIM—ticarcillin/clavulanic acid (75 µg/10 µg) (R < 16, S > 16), FOX—cefoxitin (30 µg) (R ≤ 14, 15 ≤ I ≤ 17, S ≥ 18), CTX—cefotaxim (30 µg) (R ≤ 14, 15 ≤ I ≤ 22, S ≥ 23), CAZ—ceftazidime (30 µg) (R ≤ 14, 15 ≤ I ≤ 17, S ≥ 18), FEP—cefepim (30 µg) (R ≤ 14, 15 ≤ I ≤ 17, S ≥ 18), ATM—aztreonam (30 µg) (R ≤ 15, 16 ≤ I ≤ 21, S ≥ 22), IMP—imipenem (10 µg) (R ≤ 13, 14 ≤ I ≤ 15, S ≥ 16), MEM—meropenem (10 µg) (R ≤ 13, 14 ≤ I ≤ 15, S ≥ 16), ETP—ertapenem 10 µg) (R ≤ 15, 16 ≤ I ≤ 18, S ≥ 19), DOR—doripenem (10 µg) (R ≤ 19, 20 ≤ I ≤ 23, S ≥ 24), CIP—ciprofloxacin (5 µg) (R ≤ 15, 16 ≤ I ≤ 20, S ≥ 21), AN—amikacin (30 µg) (R ≤ 14, 15 ≤ I ≤ 16, S ≥ 17), NET—netilmicin (30 µg) (R ≤ 12, 13 ≤ I ≤ 14, S ≥ 15), TOB—tobramycin (10 µg) (R ≤ 12, 13 ≤ I ≤ 14, S ≥ 15), C—chloramphenicol (30 µg) (R ≤ 12, 13 ≤ I ≤ 17, S ≥ 18), TGC—tigecyclin (15 µg) (R ≤ 14, 15 ≤ I ≤ 18, S ≥ 19).

Susceptibility testing was carried out using the disc-diffusion method, on Mueller-Hinton II Agar (bioMerieux). Cultures were incubated at 37 °C for 16–18 h. The results were interpreted according to EUCAST guidelines [52].

The double-disk synergy test and combination disk method were used to determine ESBL production. The sensitivity of the DDST can be improved by reducing the distance between the disks of cephalosporins and clavulanate. The disk approximation method was performed on a Muller-Hinton agar plate inoculated with the clinical bacterial strain, by placing disks containing CAZ—ceftazidime (30 µg), CTX—cefotaxime (30 µg) and ATM—aztreonam (30 µg) 20 mm (edge to edge) from a disk of AMC—amoxicillin/clavulanic acid (20/10 µg). Following incubation for 16–18 h at 37 °C, any enhancement of the zone of inhibition between a cephalosporin and monobactam-aztreonam disk from the amoxicillin/clavulanic acid disk, was indicative of the presence of an ESBL. *E. coli* ATCC 25922 was used as a positive control [11,53].

## 5. Conclusions

(1) The results of these experiments indicate the potential use of basil and rosemary essential oils against resistant *Escherichia coli* clinical strains, and also against extended-spectrum β-lactamase positive bacteria.
(2) The tested basil oil was more active against all *Escherichia coli* clinical strains.
(3) The action of essential oils against bacteria exhibiting different mechanisms ofresistance may beuseful, not only in treating but alsopreventingthe spreadof resistantstrains.
